# Wedge resection versus segment IVb and V resection of the liver for T2 gallbladder cancer: a systematic review and meta-analysis

**DOI:** 10.3389/fonc.2023.1186378

**Published:** 2023-07-04

**Authors:** Zhehan Chen, Jiayu Yu, Jiasheng Cao, Chenping Lin, Jiahao Hu, Bin Zhang, Jiliang Shen, Xu Feng, Win Topatana, Mingyu Chen, Haixing Fang

**Affiliations:** ^1^ The Second Clinical Medical College, Zhejiang Chinese Medical University, Hangzhou, Zhejiang, China; ^2^ Department of General Surgery, Sir Run-Run Shaw Hospital, Zhejiang University, Hangzhou, Zhejiang, China; ^3^ Zhejiang University School of Medicine, Zhejiang University, Hangzhou, Zhejiang, China; ^4^ Department of General Surgery, Second People’s Hospital of Linhai, Linhai, Zhejiang, China; ^5^ Department of Hepatobiliary Surgery, The First People’s Hospital of Fuyang, Fuyang First Hospital Affiliated to Zhejiang Chinese Medical University, Hangzhou, Zhejiang, China

**Keywords:** gallbladder cancer, wedge resection, segment IVb/V resection, prognosis, meta-analysis

## Abstract

**Objectives:**

Although guidelines recommend extended cholecystectomy for T2 gallbladder cancer (GBC), the optimal hepatectomy strategy remains controversial. The study aims to compare the prognosis of T2 GBC patients who underwent wedge resection (WR) versus segment IVb and V resection (SR) of the liver.

**Methods:**

A specific search of online databases was performed from May 2001 to February 2023. The postoperative efficacy outcomes were synthesized and meta-analyses were conducted.

**Results:**

A total of 9 studies involving 2,086 (SR = 627, WR = 1,459) patients were included in the study. The primary outcomes included disease-free survival (DFS) and overall survival (OS). For DFS, the 1-year DFS was statistically higher in patients undergoing SR than WR [risk ratio (RR) = 1.07, 95% confidence interval (CI) = 1.02-1.13, *P* = 0.007]. The 3-year DFS (*P* = 0.95), 5-year DFS (*P* = 0.77), and hazard ratio (HR) of DFS (*P* = 0.72) were similar between the two groups. However, the 3-year OS was significantly lower in patients who underwent SR than WR [RR = 0.90, 95% CI = 0.82-0.99, *P* = 0.03]. Moreover, SR had a higher hazard HR of OS [HR = 1.33, 95% CI = 1.01-1.75, *P* = 0.04]. No significant difference was found in 1-year (*P* = 0.32) and 5-year (*P* = 0.9) OS. For secondary outcomes, patients who received SR tended to develop postoperative complications (POC) [RR = 1.90, 95% CI = 1.00-3.60, *P* = 0.05]. In addition, no significant differences in intrahepatic recurrence (*P* = 0.12) were observed.

**Conclusions:**

In conclusion, SR can improve the prognosis of T2 GBC patients in DFS. In contrast to WR, the high HR and complications associated with SR cannot be neglected. Therefore, surgeons should evaluate the condition of the patients and take their surgical skills into account when selecting SR.

**Systematic review registration:**

https://www.crd.york.ac.uk/prospero/, identifier, CRD42022362974.

## Introduction

Gallbladder cancer (GBC) is a rare and fatal disease (1). Most patients affected by GBC are diagnosed at an advanced stage (2), with a 5-year survival rate of less than 5% (3, 4). Patients with T2 GBC (5, 6), as defined by the 8^th^ edition of the American Joint Committee on Cancer (AJCC) Staging Manual, must undergo surgery to improve their prognosis due to poor response to systemic therapy (7). Since it is insufficient for T2 GBC patients to receive simple cholecystectomy (8), radical cholecystectomy should be performed (9).

The most effective treatment for T2 GBC patients is extended cholecystectomy (10). Since Glenn et al. (11) proposed to combine gallbladder bed resection with cholecystectomy, it has been performed to improve the overall survival (OS) rate of GBC. Currently, wedge resection (WR) and segment IVb/V resection (SR) of the liver is the most common radical resection strategies for T2 GBC (12). WR refers to the resection of 2-3 cm of liver parenchyma from the gallbladder bed, while SR refers to the resection of the liver anatomic Couinaud’s segment IVb and V. Several studies have demonstrated significant OS or disease-free survival (DFS) of SR over WR (13, 14), whereas other studies opposed SR due to the surgical difficulties and perioperative complictions (15–20). Moreover, the surgical indications remain inconsistent. Several studies have reported that WR was recommended for T2 GBC and SR for T3 GBC (21, 22), while others believed WR was suitable for GBC invading the liver bed to a depth of under 2 cm and SR should be performed when GBC invades the liver bed to a depth of over 2 cm (23). Therefore, the optimal extent of hepatic resection for T2 GBC patients remains controversial (16, 24, 25).

In this study, we reviewed relevant studies and pooled data from multiple perspectives to compare the difference in prognosis between these two surgical procedures. This study will provide hepatobiliary surgeons with clinical guidance for selecting hepatectomy strategies for T2 GBC patients.

## Materials and methods

### Information resources and search strategy

From May 2001 to February 2023, we searched the relevant database including PubMed, Embase, Scopus, and Cochrane Library. The following free text and MeSH terms were entered into the search of the database: “gallbladder neoplasm”, “gallbladder cancer”, “hepatectomy”, “anatomic resection of liver”, “segments IVb and V”, and “wedge liver resection”. We also reviewed the references of included articles to identify additional studies.

### Inclusion and exclusion criteria

This meta­analysis conformed to the Preferred Reporting Items for Systematic Reviews and Meta-Analyses (PRISMA) statement (26). Studies that fulfilled the following criteria were included in the analysis: (a) study population: patients diagnosed as T2 GBC pathologically; (b) intervention: the studies included the comparison of WR and SR; (c) study design: randomized controlled trials (RCTs), cross-sectional study or retrospective study; (d) outcomes measure: contained at least one primary outcome (OS or DFS) or Kaplan-Meier curve.

The following studies were excluded: (a) with a Newcastle-Ottawa assessment scale (NOS) score < 4; (b) unavailability to extract or calculate necessary data from published results; (c) considerable overlap between patient cohorts; and (d) conference abstracts.

### Primary and secondary outcomes

The primary outcomes included OS and DFS. OS was defined as the duration from T2 GBC resection to death or the last follow-up. In addition, DFS was the time between surgery and tumor recurrence/metastasis or the most recent follow-up.

The secondary outcomes consisted of postoperative complications (POC) and intrahepatic recurrence (IR). POC included intraabdominal hemorrhage, respiratory dysfunction, postoperative pancreatitis, wound infection, bile leakage, cholangitis, ileus, etc.

### Data extraction and quality assessment

Two reviewers independently extracted the following variables from each study: first author, year and country of publication, study design, length of the follow-up period, the number of participants, the primary outcomes, and the secondary outcomes. We quoted the 1, 3, and 5-year survival rate and hazard ratio (HR) from the enlarged plots of the Kaplan-Meier curves. One (17) of included studies did not contain complete Kaplan-Meier curves but divided patients into two subgroups based on tumor site – hepatic and peritoneal side. To distinguish them in subsequent analyses, we labeled these two subgroups as “Araida, et al., 2009 (1)” and “Araida, et al., 2009 (2)”, respectively.

We assessed the methodological quality of all included studies, using validated NOS (27): high risk of bias (0-3 points), intermediate risk of bias (4-6 points), and low risk of bias (7-9 points). There was 100% agreement between the two reviewers.

### Statistical analyses

We performed the meta-analysis using ReviewManager (Version 5.4) and Stata (Version 16.0). We calculated the risk ratio (RR) and 95% confidence intervals (CI) to analyze the dichotomous variables. The statistical tests were two-sided, and a *P*-value ≤ 0.05 was considered statistically significant. The studies were pooled in the meta-analysis based on the Mantel-Haenszel model, which estimated the consistency of the included studies. I (2) statistic was used to assess the heterogeneity of the effect sizes. Fixed effects models were used throughout the analysis for I^2^ < 50%. When I^2^ ≥ 50%, we used random effects models.

Funnel plots, the Harbord test (for dichotomous variables), and the Egger test (for HR) were used for publication bias. The sensitivity analysis was also performed to evaluate the robustness of these results.

## Results

### The selection of trials and quality assessment

**Figure 1:**
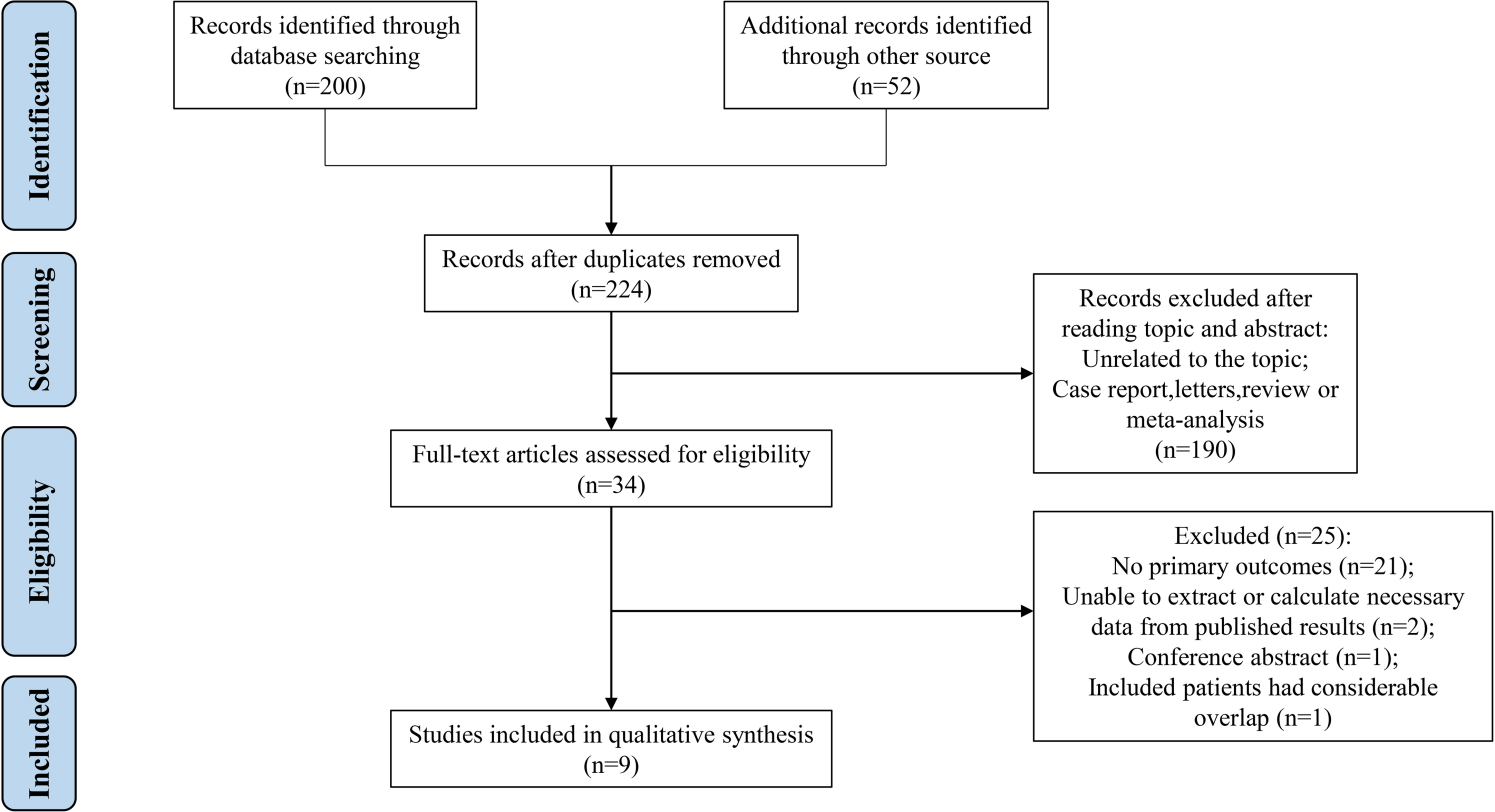
A flow diagram of the inclusion criteria of studies eligible for meta-analysis.

A total of 224 studies were initially collected, of which 190 studies were excluded after reviewing titles and abstracts. After reading the full text, we further excluded 25 studies, including 21 studies lacking primary outcomes, 2 studies with unavailable data, 1 study containing considerable overlap with another one, and 1 conference abstract. Finally, the remaining 9 studies were included in the meta-analysis (**Figure 1**). The included studies (5, 13–15, 17, 18, 28–30) were all retrospective cohort designs without RCTs. Subsequently, these patients were divided into two groups based on surgical procedures: SR (n = 627, 30.06%) and WR (n= 1,459, 69.94%). The information on authors, countries, and outcome measures of the included studies was recorded in **Table 1**. This meta-analysis comprised 1 study with an intermediate risk of bias and 8 studies with a low risk of bias. **(** **Supplementary Table 1** **)**.

**Table 1:** Demographic and study characteristics of the articles included in the meta-analysis.

Study	Period	Country	Study design	Number of patients (T2 GBC)	Surgical procedures			Outcomes
					SR		WR	
Chen, et al., 2021	2012-2016	China	Retrospective cohort	512	117		395	OS, DFS, IR, POC
Kwon, et al., 2020	1987-2017	Korea, Japan, Chile, USA	Retrospective cohort	689	257		432	DFS
Lee, et al., 2018	2001-2009	Korea	Retrospective cohort	206	45		161	OS, IR
T O Goetze, et al., 2014	1997-2014	Germany, Austria, Switzerland	Retrospective cohort	165	43		122	OS
Horiguchi, et al., 2013	1998-2004	Japan	Retrospective cohort	85	30		55	OS, DFS, POC
Wakai, et al., 2012	–	–	Retrospective cohort	51	6		45	OS
Fuks, et al., 2011	1998-2008	France	Retrospective cohort	63	39		24	OS
Araida, et al., 2009	1994-2003	Japan	Retrospective cohort	293	88		205	OS, IR, POC
Chijiiwa, et al., 2001	1983-1999	Japan	Retrospective cohort	22	2		20	OS

T2 GBC, T2 gallbladder cancer; OS, overall survival; DFS, disease-free survival; POC, postoperative complications; IR, intrahepatic recurrence.

### OS

**Figure 2:**
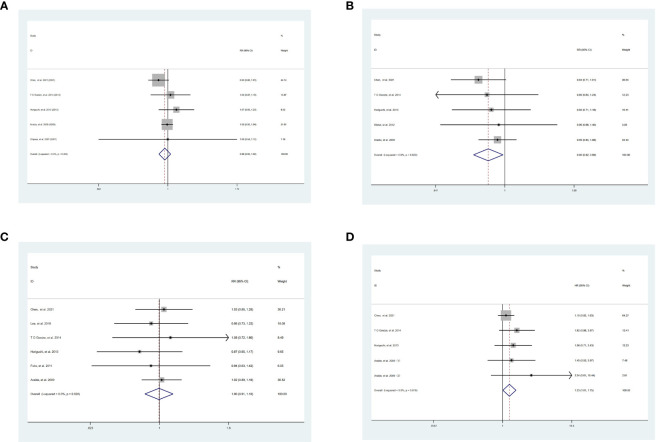
Result of the meta-analysis of OS between SR and WR, **(A)** 1-year OS. **(B)** 3-year OS. **(C)** 5-year OS. **(D)** HR of OS. SR, segment IVb and V resection of liver; WR, wedge resection of liver; OS, overall survival; HR, hazard ratio.

Although there was no significant benefit of 1-year OS between SR and WR [SR vs WR, RR = 0.98, 95% CI = 0.93-1.02, *P* = 0.32] **(** **Figure 2A** **)**, the 3-year OS [SR vs WR, RR = 0.90, 95% CI = 0.82-0.99, *P* = 0.03] was significantly lower in patients undergoing SR than those undergoing WR in the absence of heterogeneity [I^2 ^= 0.0%, *P* = 0.82] (**Figure 2B**). There was no significant difference between SR and WR of 5-year OS [SR vs WR, RR = 1.00, 95% CI = 0.91-1.10, *P* = 0.90] **(** **Figure 2C** **)**, while the HR of OS [SR vs WR, HR = 1.33, 95% CI = 1.01-1.75, *P* = 0.04] was significantly higher in SR without heterogeneity [I^2 ^= 0.0%, *P* = 0.616] (**Figure 2D**).

### DFS

**Figure 3:**
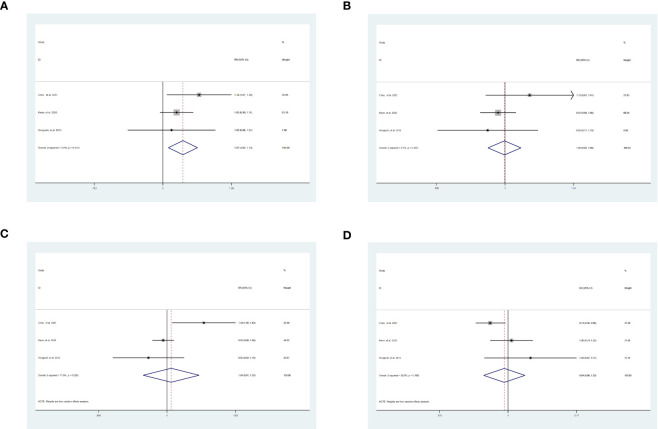
Result of the meta-analysis of DFS between SR and WR, **(A)** 1-year DFS. **(B)**3-year DFS. **(C)** 5-year DFS. **(D)** HR of DFS. SR, segment IVb and V resection of liver; WR, wedge resection of liver; DFS, disease-free survival; HR, hazard ratio.

The 1-year DFS [SR vs WR, RR = 1.07, 95% CI = 1.02-1.13, *P* = 0.007] was significantly higher in patients who underwent SR than WR without heterogeneity [I^2 ^= 0.0%, *P* = 0.41] (**Figure 3A**). The 3-year DFS [SR vs WR, RR = 1.00, 95% CI = 0.92-1.08, *P* = 0.95], the 5-year DFS [SR vs WR, RR = 1.04, 95% CI = 0.81-1.33, *P* = 0.77], and the HR of DFS [SR vs WR, HR = 0.94, 95% CI = 0.66-1.33, *P* = 0.72] were similar between the two groups (**Figures 3B-D**).

### POC and IR

**Figure 4:**
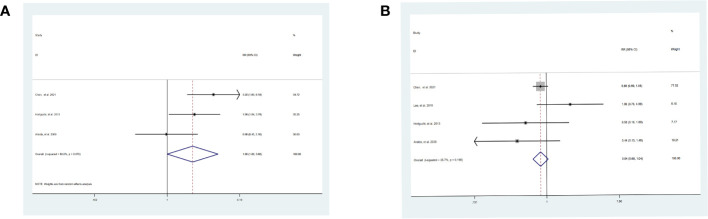
Results of the meta-analysis between SR and WR. **(A)** POC. **(B)** IR. SR: segment IVb and V resection of liver; WR, wedge resection of liver; POC, postoperative complications; IR, intrahepatic recurrence.

The POC [SR vs WR, RR = 1.90, 95% CI = 1.00-3.60, *P* = 0.05] were slightly higher in SR (**Figure 4A**). Although the heterogeneity of POC [I^2 ^= 61.0%, *P* = 0.08] was high, we used random effects models to partly eliminate the effect of this drawback. Nevertheless, the meta-analysis did not demonstrate a significant difference in IR between SR and WR [SR vs WR, RR = 0.84, 95% CI = 0.68-1.04, *P* = 0.12] (**Figure 4B**).

### Publication bias and sensitivity analysis

Regardless of study outcomes, visual inspection of the funnel plot (**Supplementary Figure 1**) and quantitative testing for most results (**Supplementary Figure 2**) revealed no publication bias. However, the HR of OS [*P* = 0.028] showed poor on the test. Sensitivity analyses confirmed the reliability of the results (**Supplementary Figure 3**).

## Discussion

This meta-analysis compared the safety and efficacy of WR and SR for T2 GBC patients using studies from 2001 to 2023. We discovered that SR could improve the prognosis of patients with T2 GBC in DFS. Notably, the high HR and complications associated with SR cannot be neglected. Therefore, surgeons should fully evaluate patients’ condition and take their surgical skills into account when selecting SR.

According to the results, SR had a slight prognostic advantage over WR for patients with T2 GBC. Despite the absence of statistically significant results from the analysis of IR, a trend indicated that SR was more advantageous. However, SR was also associated with increased survival risks and surgical difficulties compared to WR. These two contradictory findings necessitated the identification and validation of additional potential influencing factors in future research.

In the analysis, SR was associated with improved 1-year DFS, which may be attributable to the greater extent of liver resection. Sugita et al. (31) found that the veins from the gallbladder neck were connected to segment IVb, the anterior portal branch, and the right branch at the hepatic hilum. Notably, the veins of the gallbladder drained into segments IVb and V, which were exactly the extent of SR. Multiple studies have reported that SR can remove tumor micrometastasis in segments IVb and V, which may improve patients’ prognosis (23, 28, 32, 33), particularly for T2b GBC (34).

Nevertheless, the 3-year OS and HR of OS demonstrated that SR was inferior to WR. As the extent of hepatectomy enlarged, the probability of POCs increased (17, 23). Similar results were obtained in this meta-analysis. Although common complications such as intraabdominal hemorrhage and respiratory dysfunction had a limited impact on the patient’s long-term survival. Furthermore, several studies have shown that bile spillage led to widespread tumor implantation (35), resulting in increased long-term damage to patients’ prognosis. According to one (5) of the studies included in our meta-analysis, bile spillage accounted for 40% of all complications, making it one of the most prevalent complications (14, 18). In addition, the proportion of bile spillage after major hepatectomy was significantly higher than minor liver resection (36), supporting the effect of SR on bile spillage.

Lymph nodes status was one of the independent risk factors for GBC patients (14, 33, 37, 38). The probability of lymph nodes invasion (positive status) was 46.6% in T2 GBC patients (37, 39). However, we did not consider lymph nodes status in the meta-analysis, which may cause the insignificant difference in 5-year OS and DFS between SR and WR group. Notably, SR may have a significant survival advantage over WR in T2 GBC patients with negative lymph nodes status (14). As for lymph nodes drain involved in the local metastasis of GBC, it can be divided into three stations. However, lymphs nodes of the first station would drain directly to the third station without going through the second station (23). Moreover, some studies have also shown that lymphatic metastasis of GBC occurred earlier than liver invasion (6, 40, 41), leading to the limited benefits of SR. These studies demonstrated that lymph nodes status strongly influenced the prognosis of GBC patients after surgery, and we would conduct in-depth meta-analysis based on the lymphs nodes status in future studies.

Moreover, the effect of surgery on prognosis differed between T2a and T2b GBC patients (17, 34, 42). The gallbladder has two anatomically distinct sides: the inferior peritoneal and the superior hepatic side. The superior hepatic side of the gallbladder is attached directly to the liver by loose connective tissue rather than serosa, making it more susceptible to GBC invasion. In contrast, the peritoneal side of the gallbladder is not attached to the liver. Hence, extended liver resection might be more beneficial for the tumors on the hepatic side than the peritoneal side for T2 GBC (42). The patients’ prognosis was also affected by the pathological classification of GBC. Unfortunately, gallbladder adenocarcinomas, the most prevalent pathological type of gallbladder cancer (43), typically exhibited aggressive tumor biology (33).

During our literature search, we discovered several meta-analyses examining the surgical treatment for GBC patients. However, comparisons between SR and WR for T2 GBC were lacking. For instance, Burasakarn et al. (40) only compared the effect of simple cholecystectomy and radical resection, whereas Lee et al. (44) compared the effect of SR and WR for T1 GBC. Additionally, Eilard et al. (45) collected comparative information on SR and WR for T2 GBC patients without integrating the data. Therefore, it is essential to conduct a meta-analysis between SR and WR for T2 GBC.

The limitations of this study must be addressed. First, all of the included studies were retrospective, and the meta-analysis may contain selection biases and other confounding variables, such as differences in tumor biology and lymph node metastasis among the included patients. Due to the high quality of the included research and the validity of the sensitivity analysis, it was possible to mitigate several drawbacks. Second, there were insufficient data on secondary outcomes in the included studies, preventing the analysis of traditional indicators such as hospital stay, operating time, and bleeding volume. Even though the majority of outcomes lacked publication bias, some outcomes, such as the HR of OS, performed poorly in the analyses. To compare the outcomes of SR and WR for T2 GBC patients, additional high-quality retrospective studies or even RCTs with large sample sizes are necessary.

In conclusion, SR could improve the prognosis of patients with T2 GBC in terms of DFS, however taking into account a higher risk for POCs associated with such procedure. When selecting SR, the surgeons must thoroughly evaluate the clinical conditions of patients and their surgical skills.

## Data availability statement

The original contributions presented in the study are included in the article/**Supplementary Material**. Further inquiries can be directed to the corresponding authors.

## Author contributions

ZC, JY, JC, MC, and HF designed the study and collected the data; JH, BZ, JS, XF, and WT analyzed and interpreted the data; ZC, JY, JC, and MC wrote the manuscript; HF revised the manuscript; all authors made final approval of the version of the manuscript.
