# Supplementary figures and images for: Wedge resection versus segment IVb and V resection of the liver for T2 gallbladder cancer: a systematic review and meta-analysis
Source: Front Oncol. 2023 Jul 4;13:1186378. doi: 10.3389/fonc.2023.1186378 (PMC10352769; doi:10.3389/fonc.2023.1186378)

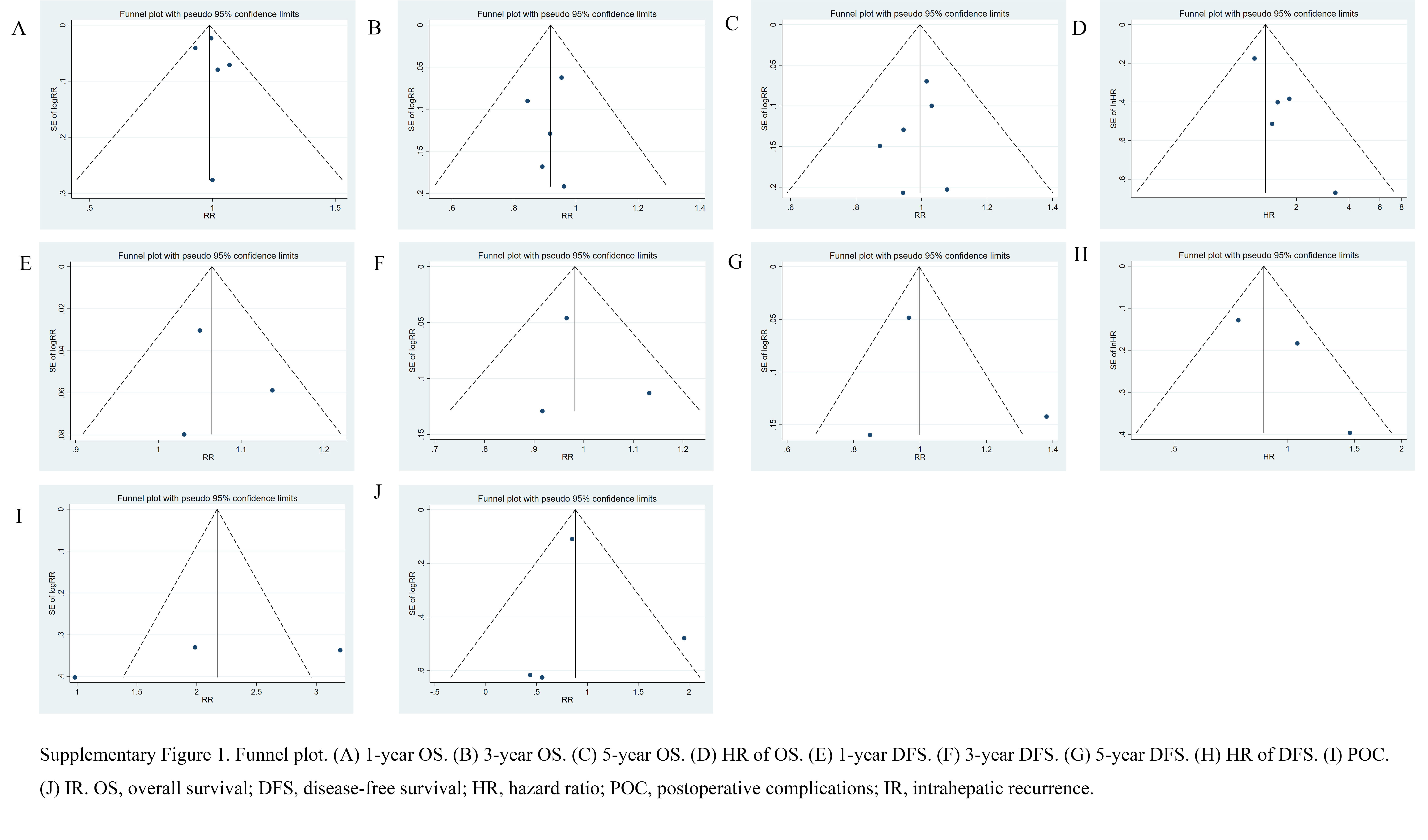

Supplement: Supplementary file 1 [file Image_1.jpeg]

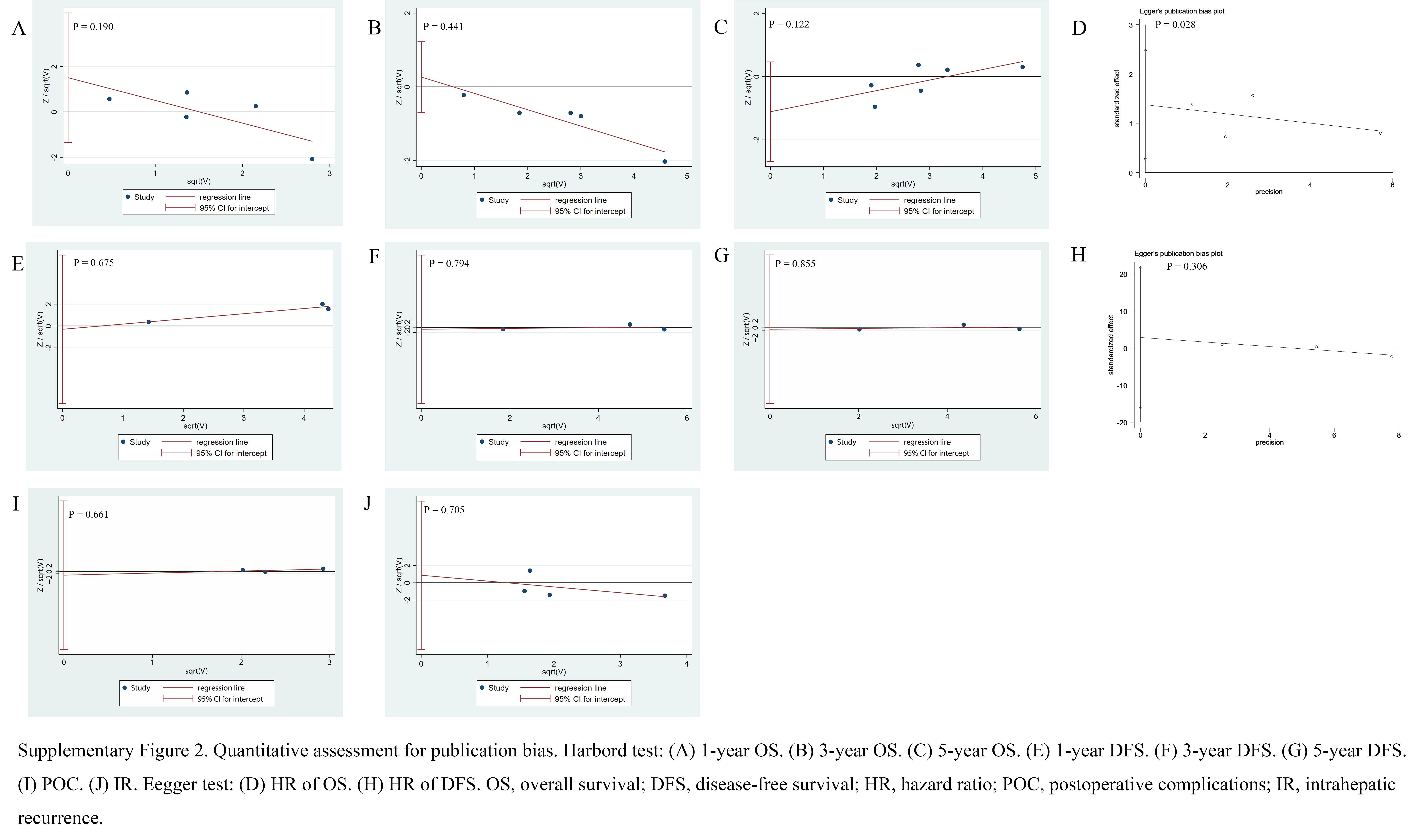

Supplement: Supplementary file 2 [file Image_2.jpeg]

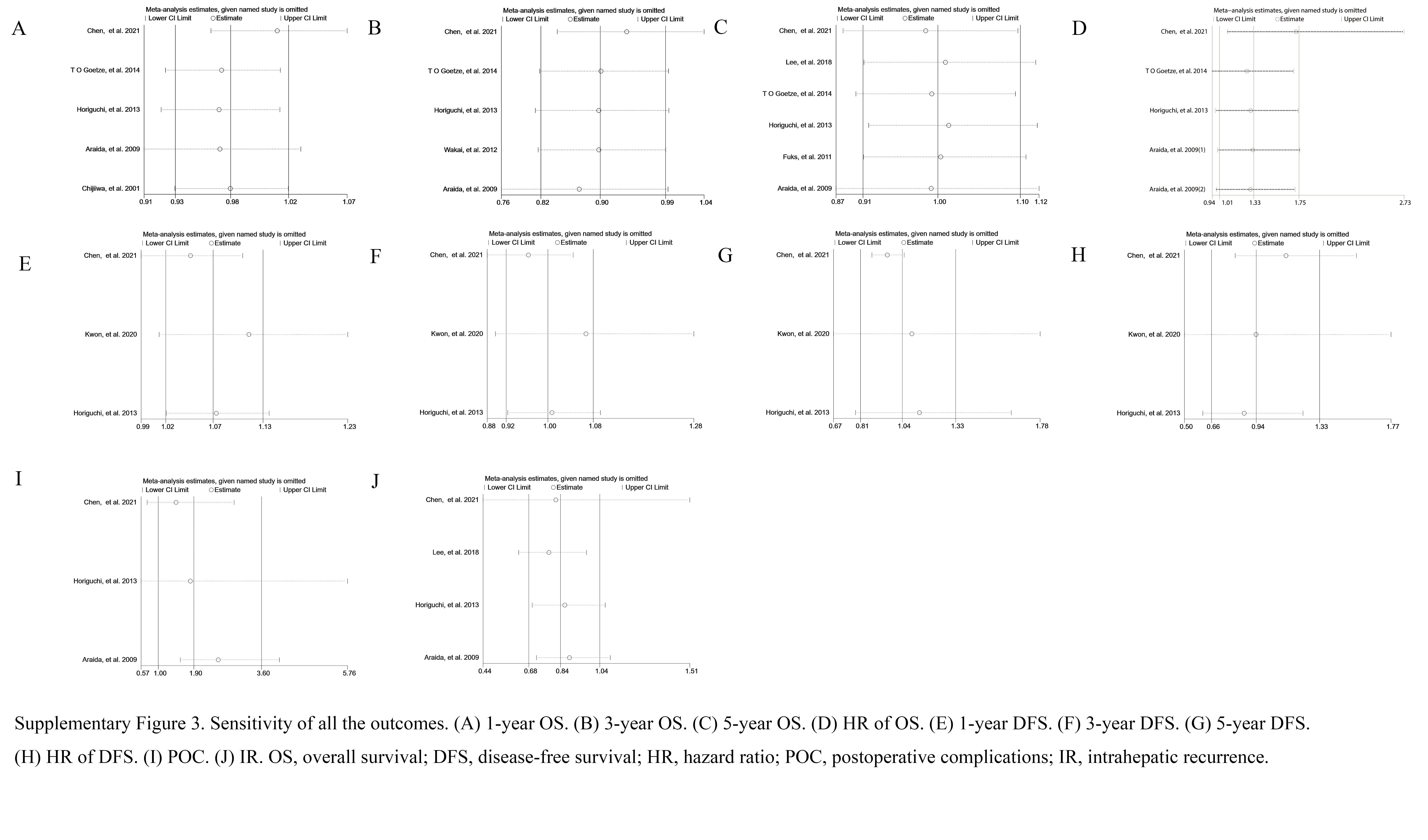

Supplement: Supplementary file 3 [file Image_3.jpeg]
