# Supplementary material for: Wedge resection versus segment IVb and V resection of the liver for T2 gallbladder cancer: a systematic review and meta-analysis
Source: Front Oncol. 2023 Jul 4;13:1186378. doi: 10.3389/fonc.2023.1186378 (PMC10352769; doi:10.3389/fonc.2023.1186378)
Supplement: Supplementary file 4 [file Table_1.docx]

**Supplementary Table 1.** Quality assessment of the included articles based on the Newcastle-Ottawa quality assessment scale for case-control studies.

| **Studies** | **Risk of bias** | **Total score** | **Selection** | | | | **Comparability** | | **Outcome** | | |
| --- | --- | --- | --- | --- | --- | --- | --- | --- | --- | --- | --- |
|  |  |  | **Exposed cohort**  **selection** | **Non‐exposed**  **cohort selection** | **Ascertainment**  **of exposure** | **Outcome was not present at**  **start of study** | **Select the most important**  **factor** | **Controls for any additional**  **factor** | **Assessment of outcome** | **Follow‐up long enough** | **Adequacy of**  **follow up of**  **cohorts** |
| (Chen, et al. 2021) | LRB | 8 | 1 | 1 | 1 | 0 | 1 | 1 | 1 | 1 | 1 |
| (Kwon, et al. 2020) | LRB | 8 | 1 | 1 | 1 | 0 | 1 | 1 | 1 | 1 | 1 |
| (Lee, et al. 2018) | LRB | 8 | 1 | 1 | 1 | 0 | 1 | 1 | 1 | 1 | 1 |
| (T O Goetze, et al. 2014) | LRB | 7 | 1 | 1 | 1 | 0 | 1 | 0 | 1 | 1 | 1 |
| (Horiguchi, et al. 2013) | LRB | 8 | 1 | 1 | 1 | 0 | 1 | 1 | 1 | 1 | 1 |
| (Wakai, et al. 2012) | ILB | 4 | 1 | 1 | 0 | 0 | 1 | 0 | 0 | 1 | 0 |
| (Fuks, et al. 2011) | LRB | 7 | 1 | 1 | 1 | 0 | 1 | 0 | 1 | 1 | 1 |
| (Araida, et al. 2009) | LRB | 8 | 1 | 1 | 1 | 0 | 1 | 1 | 1 | 1 | 1 |
| (Chijiiwa, et al. 2001) | LRB | 8 | 1 | 1 | 1 | 0 | 1 | 0 | 1 | 1 | 1 |

ILB, intermediate risk of bias; LRB, low risk of bias

Exposed cohort: patients received segment IVb and V resection of liver.

Non‐exposed cohort: patients received Wedge resection of liver.

Ascertainment of exposure: patients’ information came from reliable medical records.

Outcome was not present at start of study: patients did not receive gallbladder surgery before.

Select the most important factor: the study compared short‐term and/or long‐term outcomes between segment IVb and V resection of liver and Wedge resection of liver groups.

Controls for any additional factor: in the study, univariate and multivariate analysis was conducted to exclude the potential confounders.

Assessment of outcome: if DFS or OS was reported, it should be in the form of survival curves or 1, 3, 5‐year rates.

Follow‐up long enough: median or mean follow‐up was considered long enough at least 12 months.

Adequacy of follow up of cohorts: the loss of follow up was reported, and was lower than 20%.
